# The Care of the Eyes

**Published:** 1883-07

**Authors:** Geo. E. Blackham


### THE CARE OF THE EYES.

GEO. E. BLACKHAM, M. D.

If a man was possessed of a very valuable and delicate instrument which was absolutely necessary to him in the conduct of his business and which could only be replaced at great expense, one would naturally suppose he would use it with the greatest care, avoiding everything likely to damage it or impair its usefulness, and in case it got out of order in any way, would not allow it to be touched except by some competent instrument maker.

No man would be likely to allow a machinist or a blacksmith to open his watch and attempt to remove from its works a speck of dust which was impeding its running, lest unskillful handling should damage the delicate mechanism beyond repair. Yet, if his watch is ruined it is possible, at some expense, to replace it with another equally as good.

All this is so trite and commonplace that it seems hardly worth while to write it out.

And yet men are possessed of certain pieces of apparatus more delicate and wonderful than any watch, more necessary to comfort and usefulness, more easily damaged and, once ruined, utterly impossible to replace, but which, nevertheless, they abuse and tamper with in the most reckless fashion.

I refer to the eyes—these delicate and sensitive organs with their marvellous mechanisms for adjustment, motion, etc., are constantly subjected, by people who should know better, to most astounding abuses.

They are strained beyond endurance over fine or imperfect print, by reading with insufficient light, by recklessly gazing at too brilliant sources of illumination, by working delicate and intricate patterns of fancy work and in a thousand other ways.

If a mechanic gets a bit of metal in his eye he usually goes to some fellow workman, whom he would not permit to touch the works of his watch were they out of order, and permits him to dig away with a prick punch, a scratch awl or a bit of stick.

If a lady suffers from some form of sore eyes she goes too often the round of her lady friends, getting from this one a salve, from that one a wash and from another an alum curd, and trying a thousand and one applications, many of which are too disgusting to be named.

Then, too, there is a strange prejudice against the use of spectacles. How often does the oculist hear the old story of discomfort and even positive suffering endured for years by persons who from some error of refraction or accommodation have been obliged to strain their eyes to the utmost to see at all, and with all their efforts have had no clear and distinct vision as a reward for the pain their efforts to see without glasses have caused them; when a properly selected pair of glasses would have given clear and comfortable vision and caused the headaches and other aches to vanish, and preserved the visual organs from the deterioration which is the inevitable result of prolonged overstraining.

How often, too, do we find the professional man, lawyer or clergyman, who would not think of buying a suit of ready-made clothes lest they should not fit him to a T, purchasing a pair of ready-made spectacles, and, after a time, coming to the oculist with the sad tale, "My eyes are failing; I find that glasses don't help me, but rather makes matters worse" and upon examination it proves that the trouble is that from lack of knowledge he has made a wrong selection. The glasses are too strong or too weak, the frames do not fit, the centres of the lenses do not occupy their proper positions in relation to the pupils of the eyes, or some other defect in the grinding or mounting of the glasses renders them unsuited to his special needs.

The list of abuses to which the human eye is subjected might be extended indefinitely, but enough has been said to draw attention to an important subject, and the limits of this article will only permit of my offering a few simple suggestions for the care of these delicate organs, so useful, so necessary and so utterly impossible to replace if once spoiled.

1st. Never strain the eyes by using them with insufficient or uncertain light, or upon work so minute as to require painful strain of the accommodation.

2d. When they begin to "feel tired" give them rest. This "tired feeling" is nature's danger signal, and if not heeded trouble will follow.

3d. Beware of exposing them to too brilliant illumination. I have recently seen a case of serious damage to the eyes of a young man who had such confidence in the "strength" of his eyes that he bet he could discern the spots on the sun with the naked eye, and now has a blind spot in his visual field as the reward of his foolishness.

4th. If you find that your sight is not what it should be, if reading in the evening is getting troublesome, if the eyes weary quickly or get red and tender upon slight occasion, go to some competent oculist for advice, and if he advises spectacles, don't let pride or vanity stand in the way of accepting these important aids.

5th. If you get any foreign body, a cinder, a splinter of metal, or even an eyelash, in your eye, go to some competent medical man and have it removed at once, don't let the butcher, the baker or the candlestick maker tamper with it to the possible ruin of so delicate an organ as the eye.

6th. If your eyes get sore don't allow your friends to prescribe their white of egg, alum curds, or any other mess on the ground that it cured Mr. so and so. May be it did, but the conditions may be very different. It takes long study and careful investigation to diagnose the exact condition of an inflamed eye, and very different treatment may be needed by two sore eyes which seem to the non-professional observer to be similarly affected.

7th. Remember always that you can have but one pair of eyes, and that if you spoil these by carelessness, or recklessness, or mistreatment of any kind, the loss is irreparable, and that though, in the natural course of things, the eyes must deteriorate with advancing age, yet with proper care and the assistance of properly selected glasses, a man who has a good pair of eyes to start out with should preserve very useful vision up to or beyond the Psalmist's limit of "three score years and ten."

